# Investigating the electrochemical stability of Li_7_La_3_Zr_2_O_12_ solid electrolytes using field stress experiments†

**DOI:** 10.1039/d1ta02983e

**Published:** 2021-06-17

**Authors:** Stefan Smetaczek, Eva Pycha, Joseph Ring, Matthäus Siebenhofer, Steffen Ganschow, Stefan Berendts, Andreas Nenning, Markus Kubicek, Daniel Rettenwander, Andreas Limbeck, Jürgen Fleig

**Affiliations:** Institute of Chemical Technologies and Analytics, TU Wien Vienna Austria Juergen.Fleig@tuwien.ac.at; Leibniz-Institut für Kristallzüchtung Berlin Germany; Institute of Chemistry, TU Berlin Berlin Germany; Department of Material Science and Engineering, NTNU Norwegian University of Science and Technology Trondheim Norway; International Christian Doppler Laboratory for Solid-State Batteries, NTNU Norwegian University of Science and Technology Trondheim Norway; Graz University of Technology, Institute for Chemistry and Technology of Materials, NAWI Graz Graz Austria

## Abstract

Cubic Li_7_La_3_Zr_2_O_12_ (LLZO) garnets are among the most promising solid electrolytes for solid-state batteries with the potential to exceed conventional battery concepts in terms of energy density and safety. The electrochemical stability of LLZO is crucial for its application, however, controversial reports in the literature show that it is still an unsettled matter. Here, we investigate the electrochemical stability of LLZO single crystals by applying electric field stress *via* macro- and microscopic ionically blocking Au electrodes in ambient air. Induced material changes are subsequently probed using various locally resolved analysis techniques, including microelectrode electrochemical impedance spectroscopy (EIS), laser induced breakdown spectroscopy (LIBS), laser ablation-inductively coupled plasma-mass spectrometry (LA-ICP-MS), and microfocus X-ray diffraction (XRD). Our experiments indicate that LLZO decomposes at 4.1–4.3 V *vs.* Li^+^/Li, leading to the formation of Li-poor phases like La_2_Zr_2_O_7_ beneath the positively polarized electrode. The reaction is still on-going even after several days of polarization, indicating that no blocking interfacial layer is formed. The decomposition can be observed at elevated as well as room temperature and suggests that LLZO is truly not compatible with high voltage cathode materials.

## Abstract 2

The combination of field stress experiments with subsequent electrochemical, chemical, and structural analysis provides insight into the stability window and decomposition behavior of LLZO.

## Graphical abstract

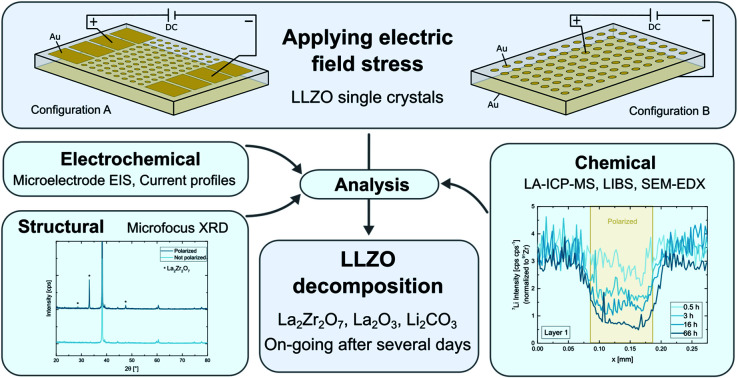

## 1. Introduction

Li-ion batteries (LIBs) are crucial for our modern society. Besides being utilized in most portable electronic devices, LIBs play an important role in emerging applications such as electric vehicles and large-scale energy storage.^1,2^ Although LIBs are well established and widely used, their application is limited by the currently employed electrolytes, which are composed of organic solvents or polymers with a dissolved Li-salt.^3^ Foremost, these liquid electrolytes are flammable causing safety issues for the batteries. Furthermore, the achievable energy density of LIBs is limited by the poor electrochemical stability of these organics, restricting the choice of electrode materials. To overcome these issues, it is of high interest to replace these organic liquid electrolytes by more stable inorganic solid ion conductors.^4^

One of the most promising solid electrolytes is cubic Li_7_La_3_Zr_2_O_12_ (LLZO), first reported by Murugan *et al.* in 2007,^5^ which is usually stabilized at room temperature by aliovalent substitution. Numerous substitution elements including Al, Ga, Nb, Ta, and Fe have been reported and Li-ion conductivities in the range of 10^−4^ to 10^−3^ S cm^−1^ have been achieved.^6–16^ Beside its high conductivity, the garnet-type solid ion conductor is known for its good electrochemical stability, particularly its chemical stability against elemental Li, enabling the use in Li metal batteries.^2,5,17,18^ However, despite intensive research on LLZO in recent years, many aspects are still not understood and several challenges remain. For example, the instability of LLZO in an ambient environment as well as interface issues can limit the application of LLZO in all-solid-state batteries.^19,20^

Also the electrochemical stability of LLZO is still an unsettled matter. Early experimental studies report a very wide electrochemical window ranging from 0 V *vs.* Li^+^/Li to at least 5 V *vs.* Li^+^/Li, implying the possible compatibility with high voltage cathode materials.^7,16,21^ In contrast to that, density functional theory (DFT) calculations show a much narrower electrochemical window of 0.05–2.91 V *vs.* Li^+^/Li.^22,23^ According to these calculations, LLZO gets oxidized at 2.91 V to form Li_2_O_2_, Li_6_Zr_2_O_7_, and La_2_O_3_.^22,23^ In another computational study based on DFT calculations, Richards and *et al.*^24^ report an oxidation potential of approx. 3.4 V *vs.* Li^+^/Li.

According to several studies, cyclic voltammetry (CV) measurements based on semiblocking electrodes, which were used in the early experimental studies,^7,16,21^ lead to an overestimation of the electrochemical stability of LLZO and are thus not suitable to determine the true electrochemical window of the material.^22,23,25^ Han *et al.*^23^ claim that the overestimation is caused by kinetic stabilization and propose the use of a Li/LLZO/LLZO-C cell for reliable CV measurements. Using this cell design, the authors determined that the oxidation of LLZO starts at about 4.0 V *vs.* Li^+^/Li.^23^ Another approach was used by Thompson *et al.*,^25^ who combined direct current (DC) chronoamperometry, alternating current electrochemical impedance spectroscopy (EIS), optical absorption band gap measurements, and first-principles calculations, leading to the conclusion that LLZO has a sufficiently large band gap of 6.4 eV to enable its use with high-voltage cathodes. Due to these controversial reports, it is obvious that more research is necessary to truly understand the stability behavior of LLZO.

In this work, the electrochemical stability of LLZO single crystals is investigated using field stress experiments in combination with subsequent electrochemical, chemical, and structural analysis. DC voltages up to 3 V were applied in ambient air using ionically blocking Au electrodes in two different geometries. In a first set of experiments, macroscopic stripe electrodes were used to conduct field stress experiments at elevated temperatures. The effects induced by the polarization were investigated using microelectrode EIS, scanning electron microscopy (SEM), as well as laser induced breakdown spectroscopy (LIBS). The revealed LLZO decomposition was further investigated using another set of experiments, in which individual microelectrodes were positively polarized against a macroscopic counter electrode. After these polarization experiments at elevated temperatures, compositional and structural changes within the material were investigated using laser ablation-inductively coupled plasma-mass spectrometry (LA-ICP-MS) and microfocus X-ray diffraction (XRD), respectively. Strong Li-depletion beneath the microelectrodes is revealed, leading to the formation of Li-poor phases like La_2_Zr_2_O_7_. The LLZO decomposition is still on-going even after several days of polarization and is also observable at room temperature, questioning if LLZO is compatible with high voltage cathode materials.

## 2. Experimental

### 2.1 LLZO synthesis

Two types of LLZO single crystals were used for the experiments: Ta stabilized samples with the nominal composition Li_6_La_3_ZrTaO_12_ (Ta:LLZO) and Ga stabilized samples with the nominal composition Li_5.8_Ga_0.4_La_3_Zr_2_O_12_ (Ga:LLZO). The single crystals were grown by the Czochralski method directly from the melt using previously dried high purity (99.99% or better) metal oxides or carbonates (in case of Li).

Ta:LLZO was grown from the stoichiometric melt of nominal composition which would naturally lead to the same composition of the grown crystal only if the compound melted congruently. This crystal was severely defective in its upper, first grown part where it contained expanded white opaque regions and many cracks. The last grown part, however, was transparent and colorless. In contrast, the Ga:LLZO was grown from a melt with 20 mol% Li_2_O excess. Also this crystals was of low quality in its first grown part and transparent with yellow color in the last part.

The powder mixtures, either stoichiometric or Li_2_O excessive, were pressed isostatically at 500 bar and sintered for 70 hours at 680 °C (Ta:LLZO) or 6 hours at 850 °C, consequently ground and pressed again at 2000 bar and sintered a second time at 1230 °C for 6 hours (Ga:LLZO). The sintered material was melted in a 40 ml inductively heated iridium crucible under protective atmosphere (N_2_ for Ta:LLZO, Ar for Ga:LLZO). In case of Ta:LLZO growth was initiated at an iridium wire that was dipped into the melt serving as a cold finger where formation of crystal nuclei was expected to occur when the melt was undercooled. For Ga:LLZO we used a roughly [100]-oriented small piece of crystal obtained in a previous experiment. In both cases, the wire, respectively the seed, was slowly pulled upwards at rates between 0.4 and 1.0 mm h^−1^ and the power of the generator was used to control the mass growth rate and therewith the diameter of the growing crystal (≈15 mm). Growth was stopped when about one third of the melt crystallized. The crystal was withdrawn from the melt and cooled down to room temperature in 15 hours.

For the investigation described in this study, samples were prepared from the transparent last parts of both crystals. The chemical composition of the synthesized samples was determined *via* ICP-OES analysis. Sample compositions of Li_6.12_La_3_Zr_0.88_Ta_1.03_O_11.9_ (normalized to 3 La pfu) and Li_6.43_Ga_0.14_La_2.84_Zr_2_O_11.68_ (normalized to 2 Zr pfu) were determined for the Ta:LLZO and Ga:LLZO crystal, respectively. Accordingly, only about one third of Ga was incorporated during crystal growth. Details on the instrumental parameters used for the ICP-OES can be found in the ESI (Table S1†). More information regarding the chemical analysis of LLZO *via* ICP-OES can be found elsewhere.^26^

Crystal slices with a thickness of about 1 mm were used for all experiments. To remove near surface reaction layers, the samples were polished by SiC grinding paper (#4000) directly before electrode preparation. Ionically blocking Au electrodes (100–200 nm thickness) were deposited by DC sputtering (MSC 010, Bal-Tec, Germany) at room temperature. Micro-structuring was performed using two different procedures. As first approach, photolithography in combination with subsequent ion beam etching was used. For the photolithography process, a negative photoresist (ma-N 1420, Micro Resist Technology, Germany) in combination with a tetramethylammoniumhydroxid (TMAH) based, aqueous-alkaline, metal ion free developer (ma-D 533/S, Micro Resist Technology, Germany) was employed. Additionally, the sample came into contact with distilled water (stopping the development process) as well as ethanol p.a. (removing remaining photoresist) during the procedure. As second approach for micro-structuring, direct sputtering using Ni shadow masks (Temicon GmbH, Germany) was applied.

**Fig. 1:**
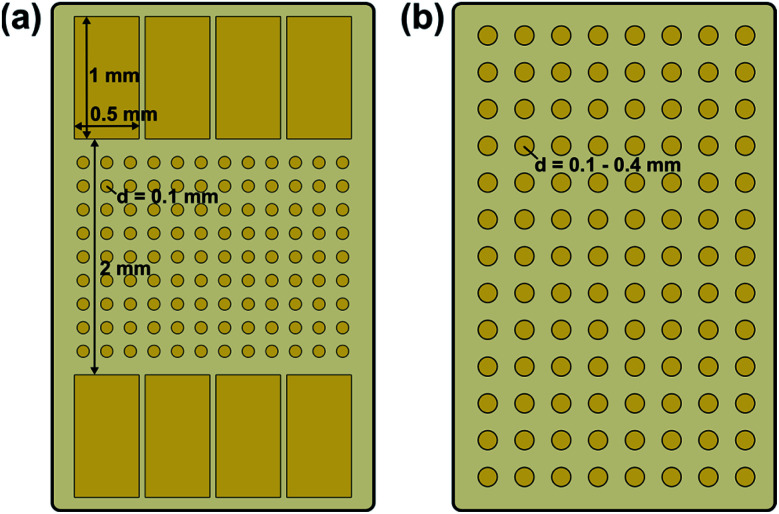
Schematic illustration of the used electrode configurations: (a) macroscopic stripe electrode with circular microelectrodes in between and (b) array of circular microelectrodes.

Two different electrode configurations were used, which are illustrated in Fig. 1. In both cases the bottom side of the samples was completely covered with an Au electrode.

### 2.2 Field stress experiments

All experiments were performed in ambient air either at elevated temperatures (350–400 °C set) or at room temperature. The sample was heated from below *via* a lab-built heating unit. Since the temperature is controlled by a thermocouple inside the heating unit, the actual sample temperature is lower than the set temperature by approx. 30–50 °C.^27^ The elevated temperature is expected to strongly accelerate decompositional phenomena due to kinetic reasons and can be regarded as a kind of ‘highly accelerated life test’ (HALT) often performed to test the stability of electrochromic devices. A 2611 Source Measure Unit (Keithley Instruments, USA) was used as voltage source and measurement unit. Au needles were employed to contact the (micro)electrodes under an optical microscope using micromanipulators to adjust the position of the needles.

#### Polarization of stripe electrodes

The impact of electric field stress on the electrochemical properties of LLZO was first investigated using the stripe electrode configuration shown in Fig. 1a. A polarization voltage of 3 V was applied at 400 °C (set temperature) *via* two opposing macroscopic stripe electrodes on the top side of a Ta:LLZO single crystal. After the polarization voltage was applied for 15 h, the sample was cooled to room temperature by switching off the heating unit. To avoid relaxation of polarization effects, the voltage was still applied during cooling.

Locally resolved EIS measurements were performed to investigate the impact of the field stress on the conductivity behaviour of the material. For that purpose, a row of microelectrodes located between the macroscopic polarization electrodes was analysed before as well as after the polarization experiment. Measurements were performed at room temperature using the Au layer on the bottom side of the sample as counter electrode. An Alpha-A high performance frequency analyser (Novocontrol Technologies, Germany) and a frequency range of 1 to 500 kHz was used for all EIS measurements. The obtained impedance spectra were fitted according to ref. 28. From the spreading resistance *R*_spread_ and the microelectrode diameter *d*, the local ionic conductivity of the probed sample volume *σ*_Me_ was calculated using eqn (1).^29^1
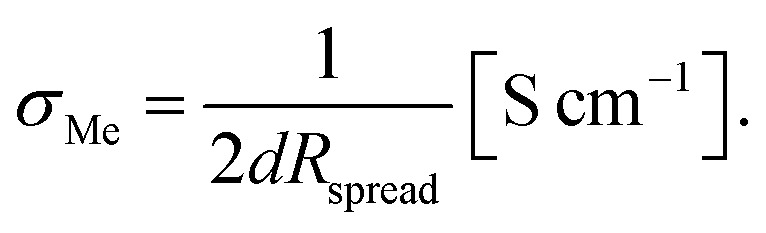


After polarization, the morphology of the electrodes was investigated *via* SEM using a Quanata 200 instrument (FEI, USA) operated at 10 kV acceleration voltage. Energy-dispersive X-ray spectroscopy (EDX) was conducted to investigate the material deposited on the cathode using an Octane Pro Silicon Drift detector (EDAX, USA) equipped on the instrument. To prevent electrostatic charging, the samples were coated with Au prior to the SEM analysis.

LIBS was used to gain spatially resolved information about the chemical composition of the sample. For that purpose, line-scans across the polarization axis were performed after the polarization experiment and EIS measurements were finished. Prior to each experiment, a pre-ablation line-scan removing the electrodes was carried out to avoid that the obtained signals are affected by the Au on top of the sample. Measurements were performed using a commercially available J200 LIBS system (Applied Spectra Inc., USA) equipped with a 266 nm frequency quadrupled Nd:YAG laser and a six-channel Czerny–Turner type spectrometer covering a wavelength range from 188 to 1048 nm. LIBS data was collected using Axiom 2.0 software provided by the manufacturer. Details on the instrumental parameters used for the LIBS measurements can be found in the ESI (Table S2†).

#### Polarization of microelectrodes

The decomposition behaviour of Ta:LLZO as well as Ga:LLZO single crystals was further investigated using the microelectrode configuration shown in Fig. 1b. Voltages up to 2.4 V were applied on individual microelectrodes using the Au layer on the bottom side of the sample as counter electrode. Field stress experiments with a stepwise voltage increase (0.2 V step size, 1.4 h holding time) as well as constant voltage measurements (2 V polarization voltage, 0.5 h–14 d holding time) were conducted, primarily at 350 °C (set temperature).

LA-ICP-MS was used to investigate field stress induced changes in the chemical composition. For the investigation, multiple line-scans across the polarized microelectrodes were performed. An untreated electrode was always investigated together with a polarized electrode and was used as reference measurement. An iCAP Qc quadrupole ICP-MS (Thermo Fisher Scientific, Germany) coupled to a NWR213 laser ablation system (ESI, USA) equipped with a 213 nm Nd:YAG laser and a fast-washout ablation cell always positioned above the actual ablation site was employed. Qtegra software provided by the manufacturer of the instrument was used for data acquisition. Prior to the experiments, the tune settings of the MS instrumentation were optimized for maximum ^115^In signal using a NIST 612 trace metal in glass standard (National Institute of Standards and Technology, USA). Detailed information about the used instrumental settings can be found in the ESI (Table S3†). The sampling depth of the experiment was determined using a DektakXT profilometer (Bruker, USA).

XRD measurements were performed using an Empyrean diffractometer (Malvern Panalytical, Germany) equipped with a focusing mirror, a 0.3 mm microfocus, and a GaliPIX3D detector. Cu Kα radiation (45 kV, 40 mA) and a 2*θ* scan range from 20° to 80° was used. For the measurements, the X-ray beam was focused on individual microelectrodes. All scans were done with a measuring time of 4.5 h per sample. The obtained diffractograms were analyzed using Panalytical Highscore.^30^

## 3. Polarization of stripe electrodes

**Fig. 2:**
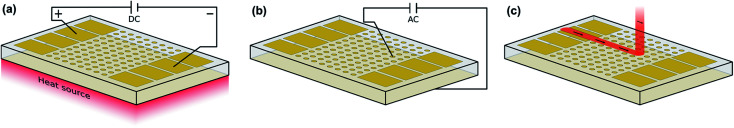
Schematic illustration of a polarization experiment with strip electrodes. (a) Application of field stress *via* two opposing macroscopic Au electrodes, (b) laterally resolved conductivity determination *via* microelectrode EIS measurements, and (c) laterally resolved chemical analysis *via* a LIBS line scan analysis.

The approach of the first type of field stress experiments is summarized in Fig. 2. A polarization voltage of 3 V was applied in ambient air at elevated temperatures (400 °C set) using macroscopic Au stripe electrodes (Fig. 2a). Before and after the polarization experiment, locally resolved conductivity measurements along the polarization axis were performed *via* microelectrode EIS measurements at room temperature (Fig. 2b). In a final step, field stress induced changes in the chemical composition were investigated using LIBS (Fig. 2c).

### 3.1 Morphology changes

**Fig. 3:**
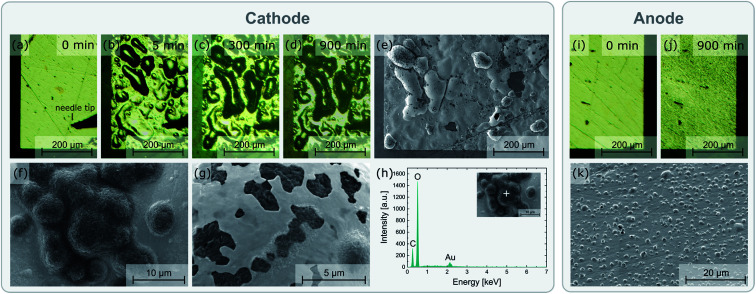
Optical microscopy images of a negatively polarized Au electrode (3 V, 400 °C set temperature) before (a), during (b and c), and after (d) the experiment. SEM images (e–g) and EDX spectrum (h) of the deposited solid, most likely Li_2_CO_3_. Optical microscopy and SEM images of the corresponding positively polarized electrode before (i) and after (j and k) the experiment, indicating gas (O_2_) formation beneath the electrode.

During the polarization experiment, the stripe electrodes undergo severe optical changes, indicating that electrochemical reactions take place. Fig. 3 shows various micrographs of the electrodes before, during, and after the polarization. At the negatively polarized electrode (cathode), a solid is deposited during the polarization. The optical appearance of the electrode changes continuously over the duration of the experiment (Fig. 3a–d), indicating an on-going reaction. SEM images reveal that a solid is formed beneath as well as on top of the Au layer (Fig. 3e–g). Most likely, Li_2_CO_3_ or another Li-containing salt (LiOH, Li_2_O) is formed due to the reduction of O_2_ from air in presence of CO_2_ and H_2_O, *e.g.*:2Li^+^ + 2e^−^ + CO_2_ + ½O_2_ → Li_2_CO_3_

Accordingly, oxygen is reduced at this electrode. This is confirmed by the EDX spectrum of the deposited solid (Fig. 3h). Beside Au, most likely originating from sample coating prior to the SEM analysis, only C and O can be observed. This shows that the deposited substance does not contain La, Zr, or Ta, leaving only Li as a possible cation (Li cannot be detected by conventional EDX). In principle, this electrochemical reaction is expected to occur close to the triple phase boundaries. The fact that large parts of the electrode surface are covered by reaction products indicates that the Au layer becomes porous during the field stress experiment. Another possible option is that Li_2_CO_3_ (or another Li-containing salt) is not formed directly, but initially metallic Li or an Li/Au alloy accumulates at the cathode, which is then converted to Li_2_CO_3_ due to reaction with the ambient environment. Strong alloying, however, should cause a severe shift of the Li chemical potential in the electrode, which is not confirmed by the polarization experiments performed on microelectrodes shown later in this work (*cf.* Section 4). Hence, we suppose that some defects in the Au films (cracks, *etc.*) allow direct access of gas phase to the LLZO surface also ‘within’ the electrode and thus enable growth of Li-containing salts. This may cause further morphological changes of the electrode and further growth.

The positively polarized electrode (anode) shows a rough surface after polarization (Fig. 3i–k). The roughness appears to be caused by gas bubbles, possibly arising from O_2_ formation due to oxidation of oxide ions, lifting parts of the electrode from the sample surface. To compensate the occurring loss of O^2−^ ions, negatively charged Li vacancies need to be created, ultimately leading to either (1) LLZO with a sub-stoichiometric amount of O^2−^ and Li^+^:Li_7_La_3_Zr_2_O_12_ → Li_7−2*x*_La_3_Zr_2_O_12−*x*_ + *x*/2O_2_ + 2*x* Li^+^ + 2*x* e^−^

Or (2) the formation of Li-poor phases such as La_2_Zr_2_O_7_ and La_2_O_3_: 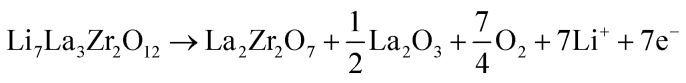


The substitution element is not considered in the given reaction equations for simplicity reasons. Additional decomposition products might be formed due the presence of a dopant (*e.g.*, LaTaO_3_ in case of Ta). The formation of La_2_Zr_2_O_7_ due to electric field stress is confirmed by XRD later in this work (see Fig. 10).

The measured current flowing through the sample during polarization (see ESI, Fig. S1†) is also in accordance with our assumption of a continuous electrochemical reaction. After a rapid decrease within the first minutes of the experiment, the current stabilized and remained at approx. 1 μA for the rest of the measurement. Given the electrode and sample geometry (distance between electrodes = 2 mm; cross section = 0.5 mm^2^), an electronic conductivity of about 1.3 × 10^−5^ S cm^−1^ would be necessary to reach such a high steady-state current, which is unlikely considering the values reported in literature (5 × 10^−12^ to 2 × 10^−9^ S cm^−1^ at room temperature^14,25,31^ and in the range of 10^−7^ S cm^−1^ at 350 °C (ref. 32)). We therefore attribute the measured current to continuous decomposition of the sample caused by field stress, and thus largely Li^+^ current.

### 3.2 (Electro)chemical analysis

**Fig. 4:**
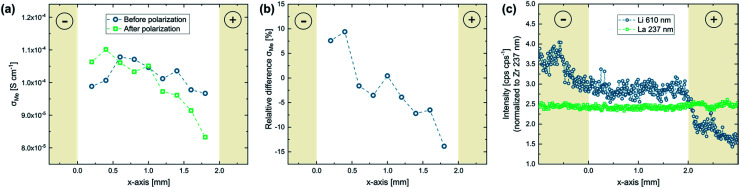
Locally resolved investigation of the effects of a stripe electrode polarization experiment (3 V, 400 °C set temperature): (a) local conductivity before and after the experiment probed *via* microelectrode EIS measurements, (b) relative conductivity change for the individual electrodes, and (c) chemical analysis at and between the electrodes measured *via* LIBS. The results show significant changes of the conductivity behavior between the electrodes as well as strong Li stoichiometry variations at the electrodes, confirming that an electrochemical reaction takes place.

In Fig. 4 the impact of the field stress on the conductivity behavior as well as the chemical composition of the material is visualized. Fig. 4a shows the local conductivity along the polarization axis before and after the bias was applied. The local conductivity measurements were performed *via* microelectrode EIS measurements using the Au layer on the bottom side as counter electrode (see Fig. 2b). To better illustrate the impact of the polarization, the relative differences of these two conductivity measurements are shown in Fig. 4b. Since most of the voltage between a microelectrode and a macroscopic counter electrode drops very close to the microelectrode, only the sample volume close to the microelectrode is probed by the measurement, thus providing laterally resolved information.^33^ Typical impedance spectra found in these measurements are shown in the ESI (Fig. S2†). The charge transport in the probed sample corresponds to the high frequency arc visible in the spectra, which is described by a resistive element (*R*_spread_) in the equivalent circuit. Detailed information regarding the evaluation of microelectrode EIS measurements performed on LLZO can be found elsewhere.^28^

The microelectrode measurements reveal a clear impact of the polarization experiments on the conductivity behavior of the material. While the local ionic conductivity increased close to the cathode, the opposite effect can be observed close the anode: the conductivity decreased up to 15% (see Fig. 4a and b). Interestingly, this effect cannot be correlated to changes in the Li stoichiometry, since chemical analysis *via* LIBS did not reveal any variations of the Li-content between the electrodes (Fig. 4c). The changes in the conductivity behavior were therefore either caused by Li stoichiometry changes too small to be observed *via* our LIBS measurements, or by other factors like local variations of site occupancies or oxygen vacancies.^34^ However, in contrast to the region between the electrodes, the LIBS analysis revealed huge variations in Li very close and/or beneath the stripe electrodes with almost no change in La. While an increased Li content can be observed on the cathode, most likely due to the formation of Li_2_CO_3_ as already confirmed by EDX (see above), the Li concentration strongly decreased at the anode, confirming the presence of Li-poor phases and/or LLZO with a Li^+^ sub-stoichiometry. The results thus confirm that the applied electric field stress leads to an electrochemical reaction decomposing the material.

**Fig. 5:**
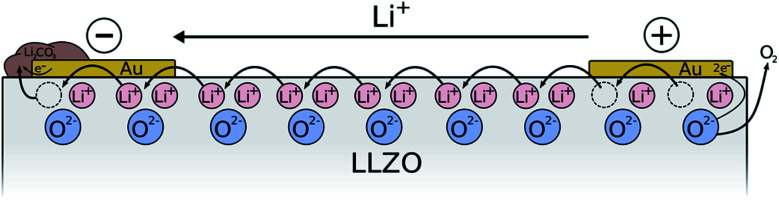
Schematic illustration of the processes induced by LLZO polarization *via* stripe electrodes. Li-containing salts like Li_2_CO_3_ are deposited at the cathode due to the reduction of O_2_ from air in presence of CO_2_ and H_2_O. On the anode, O_2_ is formed due to oxidation of oxide ions, leading to LLZO with sub-stoichiometric amount of O^2−^ and Li^+^ and/or Li-poor phases. During the reaction, Li-ions are constantly transported from the anode to the cathode.

To summarize our findings, the processes taking place during sample polarization are visualized in Fig. 5. As already discussed, O-ions of LLZO are oxidized and O_2_ from the surrounding air is reduced anode and cathode, respectively. Overall, Li-ions are transported through the sample, first leaving vacant Li^+^ sites as well as O-vacancies in LLZO at the anodic side, and ultimately leading to the formation Li-poor phases like La_2_Zr_2_O_7_.

Please note: the conducted experiments show that LLZO decomposes when a bias of 3 V is applied at 400 °C, but this does not mean that the electrochemical window of LLZO is <3 V. The reason for this is that the Li chemical potentials of neither the cathode nor the anode is fixed, since ionically blocking Au electrodes are used on both sides. We also do not have sufficient information so far, which impurity levels are allowed in inert gases to avoid this process.

## 4. Polarization of microelectrodes

**Fig. 6:**
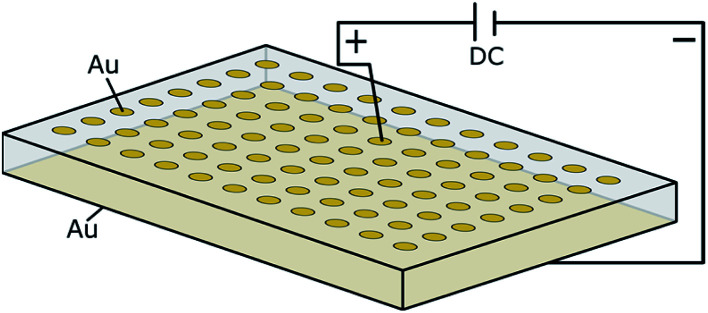
Schematic illustration of a polarization experiment using microelectrode configuration. Au microelectrodes with a diameter ranging from 100 to 400 μm were utilized. A Au layer covering the whole bottom side of the sample is used as counter electrode.

To investigate the decomposition behavior of LLZO under electric field stress in more detail, individual Au microelectrodes on Ta- as well as Ga stabilized single crystals were positively polarized in ambient air under various conditions. The used measurement setup is visualized in Fig. 6. Since the area of the macroscopic Au counter electrode is several orders of magnitude larger than the area of the microelectrode, only very minor material changes are expected at the macroscopic electrode. Therefore, we assume that the chemical potentials of LLZO at the counter electrode does not vary significantly during polarization despite its ionically-blocking character, making the used configuration somehow comparable to the Hebb–Wagner polarization technique with one reversible electrode. This, however, requires that again Li-containing salts are formed at the counter electrode rather than metallic Li or Li/Au alloy. This assumption is supported be several observations: first, the open-circuit voltage (OCV) between an untreated microelectrode and the counter electrode does not change even after several polarization experiments. Second, the current voltage curves of the microelectrodes do not depend on the number of subsequent polarization experiments with other microelectrodes. Third, Li alloying at the Au electrode (instead of Li-salt formation) should strongly reduce the local Li chemical potential to values of 1 V or lower with respect to Li^+^/Li and our results on stability limits (see below) would become very unrealistic. Moreover, such Li/Au alloy should not be stable at our experimental conditions (350 °C and air).

### 4.1 Stepwise voltage increase

**Fig. 7:**
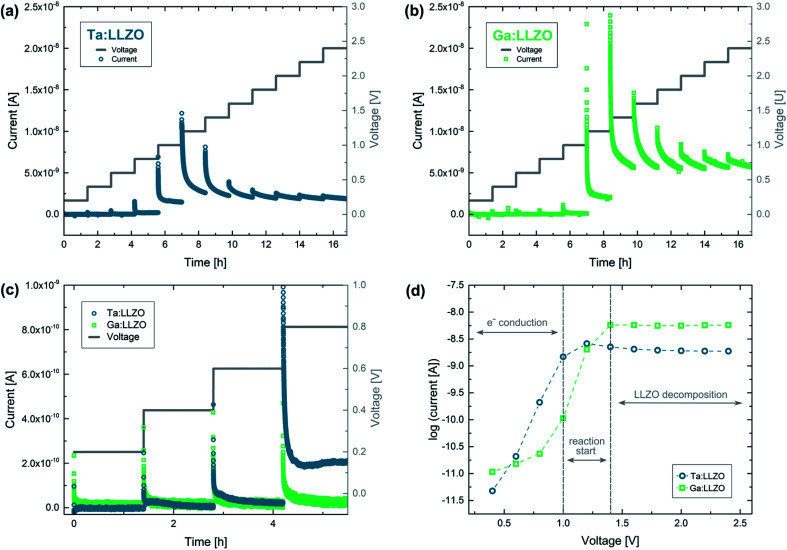
Polarization of Au microelectrodes with stepwise voltage increase (0.2 V step size, 1.4 h holding time, 350 °C set temperature, 100 μm electrode diameter). Current–time profiles for (a) Ta:LLZO and (b) Ga:LLZO single crystals as well as (c) a zoomed-in plot for the lowest voltages and (d) the ‘stabilized’ currents at the end of each voltage step, plotted on logarithmic scale. After increasing until 1.2–1.4 V, the measured currents stay constant for higher voltages, indicating a voltage independent decomposition reaction in this range.

In a first set of experiments, which were performed at elevated temperature (350 °C set), the applied voltage was stepwise increased until a final voltage of 2.4 V was reached. Fig. 7 shows the results of such measurements for a Ta- as well as a Ga stabilized LLZO single crystal. For both samples, a strong current increase can be observed at the first few voltage steps. Interestingly, the current behavior changes drastically at 1.2 V and 1.4 V for Ta:LLZO (Fig. 7a) and Ga:LLZO (Fig. 7b), respectively. The current stops increasing with the applied voltage and instead becomes more and more voltage independent for the later voltage steps. This significantly different behavior in the two different voltage regimes indicates that fundamentally different processes are limiting the charge transfer. This hypothesis is further confirmed by the time-dependencies within the individual voltage steps, also showing significantly different behavior: while in the low-voltage regime the current decreases very fast at the beginning of each voltage step, quickly leading to a constant ‘steady-state’-type current (Fig. 7c), the current decrease is relatively flat in the high-voltage regime.

To better visualize the relationship to the applied voltage, the measured currents for each voltage step are plotted on a logarithmic scale in Fig. 7d. In this plot, the currents measured at the end of the corresponding voltage step are shown, representing the ‘stabilized’ current obtained when voltage is applied for a certain time. It becomes clear from the time dependencies in Fig. 7a and b that this does not represent a true steady-state current for the high-voltage regime, but the prevailing trends are well accessible. Interestingly, an almost exponential current increase can be observed for the lower voltages (0.2–1.0 V). The origin of the steady-state current in the regime has not been investigated in detail so far. However, electron conduction is a likely explanation with realistic conductivity values in the 10^−9^ S cm^−1^ range. In the high-voltage regime (1.4–2.4 V), the measured currents are nearly constant for each voltage step, once more confirming the distinctly different conductivity behavior for higher voltages. Constant voltage experiments presented later in this work reveal that LLZO decomposition is the main source of current if a voltage of 2 V is applied on the material. Most probably the entire constant current regime is characterized by the same electrochemical process and thus we suggest that already at 1.2–1.4 V LLZO decomposition takes place. The two different regimes (electron conduction and LLZO decomposition) are also indicated in Fig. 7d.

To make any conclusion about the electrochemical stability window of the material, it is necessary to know the chemical potential of Li at the ionically blocking counter electrode, which hardly changes in this experiment (see above), but is not truly well defined in our case. This chemical potential has to be estimated. Since during synthesis of the single crystals the material is surrounded by Li_2_O in the gas phase, the chemical potential of Li in LLZO is very likely defined by Li_2_O (around 2.9 V *vs.* Li^+^/Li (ref. 35)). Accordingly, also the counter electrode is at 2.9 V *vs.* Li^+^/Li. From our experiments we can thus conclude a stability limit of 4.1–4.3 *vs.* Li^+^/Li at this elevated temperature. These finding are in agreement with the experimental data of Han *et al.*^23^ (LLZO oxidation starts at about 4.0 V *vs.* Li^+^/Li at room temperature), and support the hypothesis that extended electrochemical window observed in other experimental studies originates from kinetic stabilization.^22,23^

### 4.2 Constant voltage

To further investigate the decomposition behavior of LLZO, individual Au microelectrodes were polarized with constant voltage for different time intervals (0.5–66 h). 2 V was chosen as fixed voltage, since (1) it ensures a maximum decomposition rate (*cf.*Fig. 7), and (2) corresponds to 4.9 V *vs.* Li^+^/Li according to our estimation of the counter electrode's chemical potential and therefore simulates the typical voltage range of high-voltage lithium (ion) batteries. Like in the previous experiments, the electric field stress was applied at elevated temperatures (350 °C set) to enhance the kinetics of the LLZO decomposition. A typical current profile of such a polarization is shown in the ESI (Fig. S3†). The measured current decreases over time but stays relatively high (>0.5 nA, *i.e.* a current density of 6 μA cm^−2^ with respect to the microelectrode) even after several days of polarization, indicating an on-going electrochemical process. Translating the final current to a hypothetical bulk resistivity *via* the spreading resistance formula (eqn (1)) we get a value of 1.5 × 10^−8^ S cm^−1^. Electron conduction is thus a feasible candidate for the remaining current. However, in the following we show that a significant part of this current is ionic.

#### Chemical analysis

**Fig. 8:**
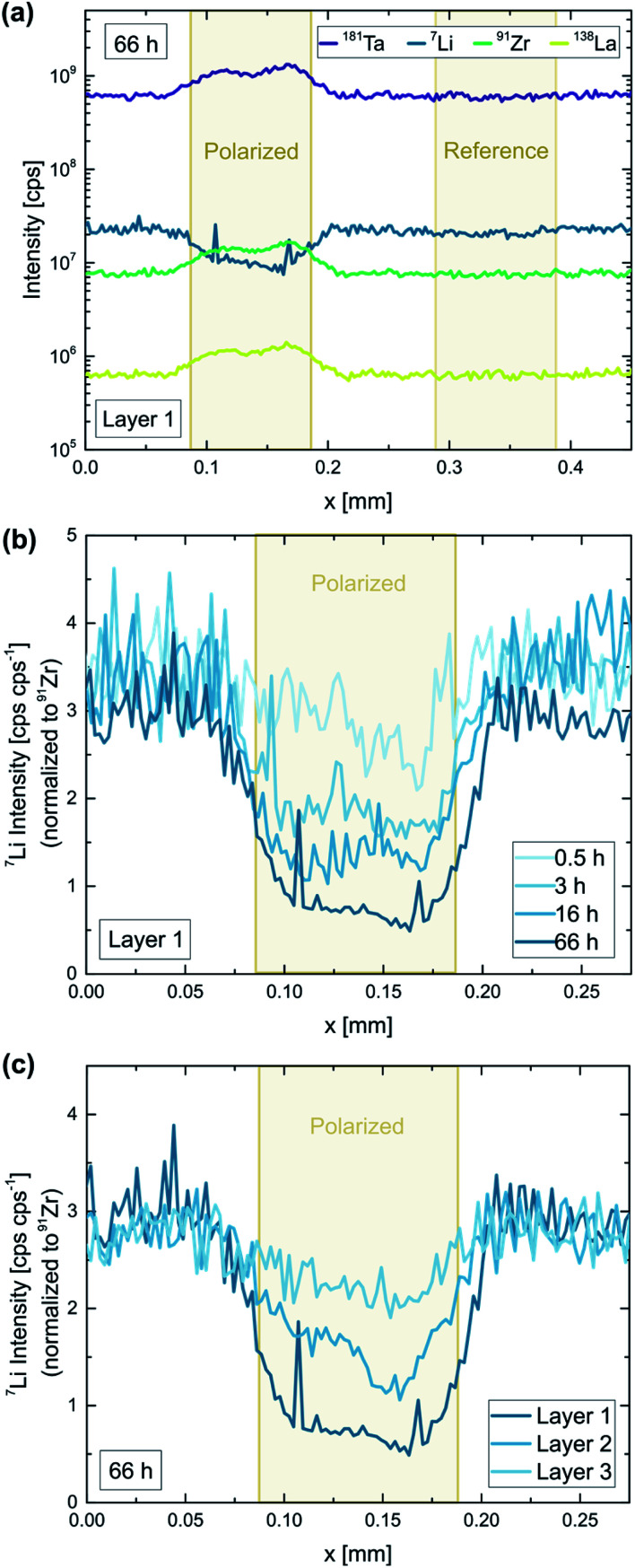
LA-ICP-MS analysis of a Ta:LLZO single crystal after a constant voltage experiment (2 V voltage, 0.5–66 h polarization time, 350 °C set temperature, 100 μm electrode diameter). (a) Raw signal intensities corresponding to a polarized (66 h) as well as an untreated electrode. Normalized Li signal for (b) different polarization times and (c) subsequent ablation passes. Significant Li-depletion beneath the electrode is induced by the applied field stress, getting more pronounced even after several days of polarization.

After the polarization experiment, the chemical composition beneath the electrodes was probed *via* LA-ICP-MS. Fig. 8 shows the results of such an LA-ICP-MS analysis for a Ta:LLZO single crystal. Significant changes of the chemical composition can be observed, confirming that LLZO decomposition was induced by the applied voltage. This is in agreement with the LIBS analysis shown in the previous section, also revealing stoichiometry changes due to electric field stress (*cf.*Fig. 4c). For the LA-ICP-MS analysis, line-scans across the microelectrodes were used, whereas each line-scan covered one polarized as well as one untreated electrode. The untreated electrodes served as reference, showing that the analyte signals are not affected by the Au electrode on top of the sample. In contrast to that, a significant decrease of the Li signal can be observed when a polarized electrode is probed, which corresponds to a Li-depletion beneath the electrode. Interestingly, also all other analytes seem to be affected by the polarization, since the signals of La, Zr, and Ta signals increase at the electrode (Fig. 8a). However, the signal ratios of La, Zr, and Ta stay constant, meaning that only Li-stoichiometry was changed by the polarization. Most likely, the applied field stress induced phase changes which altered the ablation behaviour of the material, thereby affecting the signal intensities of all analytes.

In Fig. 8b and c, the obtained Li signal is normalized to the intensity of the corresponding Zr signal. In the normalized signal, variations in material ablation during the measurement are compensated, making it a good representation of the Li-stoichiometry. A decrease of up to 83% can be observed for the longest polarization time (66 h), confirming that Li-ions are strongly depleted in the topmost sample layer beneath the electrode. Comparing different polarization times shows that the observed Li-depletion steadily increases over time (Fig. 8b). This indicates that the induced LLZO decomposition is on-going even after several days of polarization and is not stopped by the formation of an interface layer.

To investigate effects deeper inside the material, each electrode was analyzed two more times after the initial LA measurement. Each ablation pass removed approx. 2 μm material, giving access to (rough) depth-resolved information. In Fig. 8c, the three measurements of the longest polarized electrode (66 h) are compared. The induced Li-depletion is less pronounced for every subsequent ablation pass, however, even for the third and last sample layer significant effects (30% decrease) can be observed. This means that even in a sample depth of approx. 4–6 μm material changes have been induced by the applied field stress. Given the fact the effect is relatively small for the third layer, and is not observable at all for electrodes with lower polarization times, we still can assume that most of the affected sample volume is probed by the analysis.

To investigate if the relatively high currents during electrode polarization were indeed caused by the observed Li-depletion, the total amount of transported Li-ions Li^+^_trans_ was estimated using the measured stoichiometry changes. The formula2
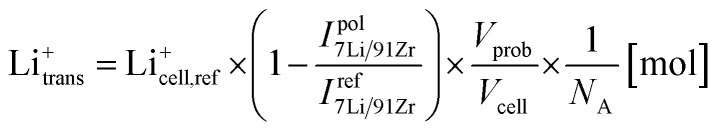
was used for the calculation, where Li^+^_cell,ref_ is the number of Li-ions in the cubic LLZO unit cell considering the sample stoichiometries determined *via* ICP-OES (49.0 Li^+^ Ta:LLZO, 51.4 Li^+^ Ga:LLZO), *I*^pol(ref)^_7Li/91Zr_ the normalized Li intensity of the polarized (reference) microelectrode [cpscps^−1^], *V*_prob_ the probed sample volume [m^3^], *V*_cell_ the volume of a cubic LLZO unit cell (2.188 × 10^−27^ m^3^ (ref. 36)), and *N*_A_ Avogadro constant (6.022 × 10^23^ mol^−1^). *V*_prob_ was calculated based on the crater depth and the electrode size, assuming that the Li-ion transport occurs uniformly beneath the electrode. The probed sample depth for the individual line scans was defined using the La signal of the analysis.

**Fig. 9:**
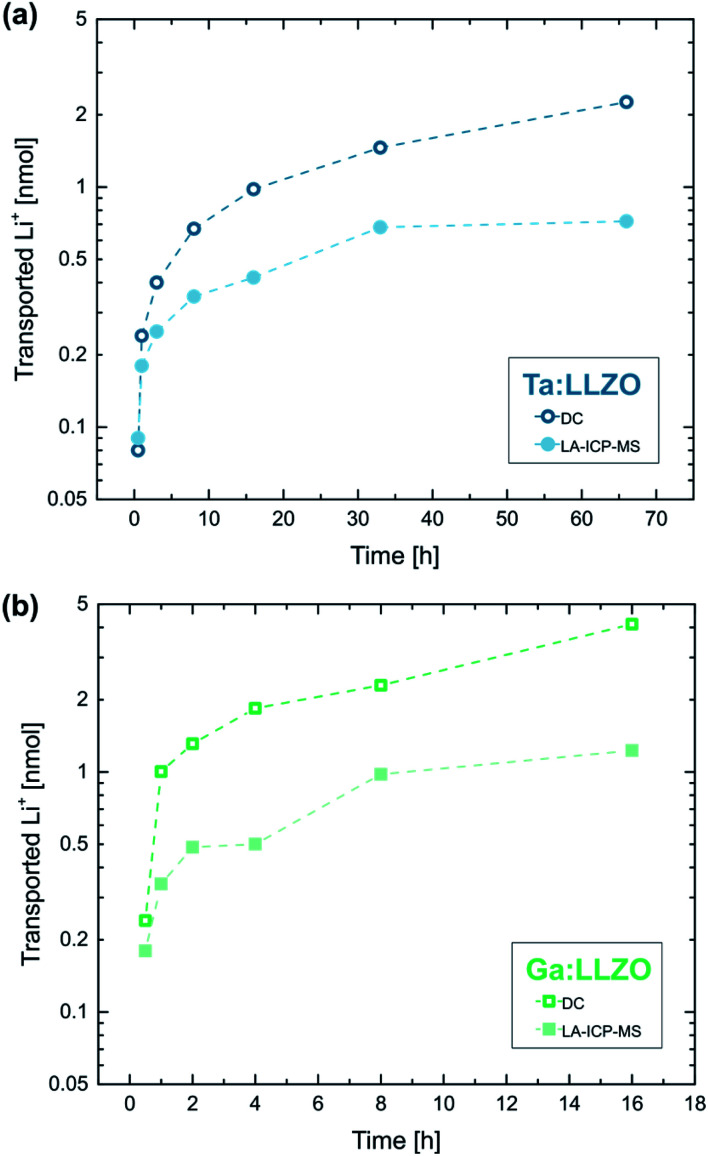
Total amount of transported Li-ions during electrode polarization (2 V voltage, 350 °C set temperature, 100 μm electrode diameter) determined *via* LA-ICP-MS as well as DC measurements, for (a) Ta:LLZO (0.5–66 h) and (b) Ga:LLZO (0.5–16 h polarization time) single crystals. The values obtained from the current profiles are generally higher but are in the same order of magnitude even for the longest polarization experiments, confirming that most of the polarization current is caused by LLZO decomposition.

Fig. 9a shows the calculated amounts of transported Li-ions for the already discussed polarization series on Ta:LLZO (*cf.*Fig. 8). The LA-ICP-MS determination is compared to values obtained from the corresponding current measurements, calculated under the assumption that all measured current solely originates from irreversible transport of Li-ions. The values obtained from the current profiles are generally higher and the relative difference increases with increasing polarization time. However, the results of both quantification approaches show a very similar trend and are in the same order of magnitude even for the longest polarization experiments. This reasonable agreement thus confirms that a substantial part of the polarization current is indeed caused by LLZO decomposition and the associated Li-depletion beneath the anode. The deviation between the values obtained by LA-ICP-MS and DC measurements might be caused by several reasons, namely (1) contribution of other processes (*e.g.*, electronic conduction) to the total current, (2) inaccuracies of the assumptions used for the calculations (*e.g.*, non-uniform Li-ion transport beneath the electrode), and (3) additional Li-depletion deeper in the material not probed by the LA analysis.

An analogue series of polarization experiments performed on a Ga:LLZO single crystal shows very similar results (Fig. 9b). The total amount of transported Li-ions is significantly higher compared to Ta:LLZO, which is in agreement with the higher decomposition current found in the polarization experiments with stepwise voltage increase (*cf.*Fig. 7). Beside that, the only observable difference was a higher ablation rate for Ga:LLZO during the LA experiment (about 3 μm per ablation pass). Overall, the substitution element only seems to affect the decomposition rate, not the process itself.

While in all previously shown experiments micro-structuring was performed using a combination of photolithography and ion beam etching, the microelectrodes used for the experiment series on Ga:LLZO were prepared *via* direct sputtering using a Ni shadow mask. Since this process is solvent-free, it can be excluded that contact with protic solutions during photolithography (see Experimental), potentially leading to Li^+^/H^+^ exchange,^37–39^ is the reason for the observed phenomena.

#### Structural analysis

**Fig. 10:**
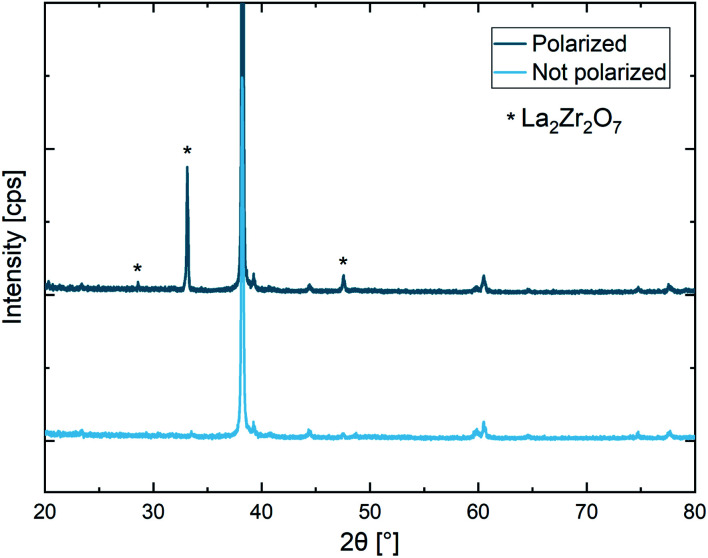
Microfocus XRD measurement after a constant voltage polarization experiment (2 V voltage, 120 h polarization time, 350 °C set temperature, 400 μm electrode diameter). La_2_Zr_2_O_7_ was formed due to applied electric field stress.

Due to the strong Li-depletion beneath the electrode, (*cf.*Fig. 8), it is to be expected that Li-poor phases are formed during the polarization. To investigate these structural changes, polarized electrodes were investigated using microfocus XRD and compared to pristine electrodes (Fig. 10). Additional reflexes can be observed in the diffractogram, showing that La_2_Zr_2_O_7_ was formed during the experiment. The results confirm the decomposition reaction proposed in Section 3.1: at the anode O^2−^ is oxidized, leading to the formation of La_2_Zr_2_O_7_ and La_2_O_3_. Additionally, also LLZO with a sub-stoichiometric amount of O^2−^ and Li^+^ might be formed to some extent.

**Fig. 11:**
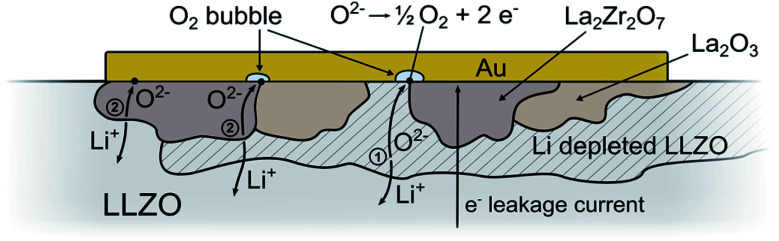
Schematic drawing of LLZO decomposition beneath a positively polarized Au electrode including potential reaction paths. La_2_Zr_2_O_7_ and La_2_O_3_ are formed due to the oxidation of O^2−^, which might be accompanied by the formation of LLZO with a sub-stoichiometric amount of O^2−^ and Li^+^ (Li_7−2*x*_La_3_Zr_2_O_12−*x*_) as intermediate product. To ensure continuous depletion of Li, oxide ion conduction in LLZO (path 1) or in a decomposition product (path 2) is necessary. In addition to the electrochemical reactions, also a certain e^−^ leakage current across the entire sample might contribute to the total charge carrier transport.

This also raises the question of the species carrying the current across this zone with decomposition products. This is certainly strongly dependent on the exact 3D distribution of the reaction products in this reaction zone. We hardly expect a simple layer-by-layer structure. However, we may face a situation where not only Li^+^ transport but also oxide ion transport and electron transport may play a role (see sketch in Fig. 11). Owing to the lack of reducible cations, Li-depletion in LLZO most probably takes place *via* formation of oxygen vacancies. Substantial oxygen vacancy concentrations and O^2−^ conduction in LLZO was already confirmed in ref. 34. Any further depletion of Li within an LLZO phase (forms Li_7−2*x*_La_3_Zr_2_O_12−*x*_) thus requires oxide ion conduction in LLZO (path 1 in Fig. 11). Depletion of Li in LLZO without direct contact to the electrode requires further conduction of O^2−^ also in a reaction product (path 2). In parallel to these faradaic currents with electrochemical reactions we may have a certain e^−^ leakage current across the entire sample. However, the LA-ICP-MS measurements clearly showed existence of substantial faradaic processes which do not stop due to the limited O^2−^ conduction in the reaction zone even after 5 μm thick layers of reaction products have formed.

In summary, these experiments clearly show that significant LLZO decomposition is induced at an applied voltage of 2 V, which corresponds to approximately 4.9 V *vs.* Li^+^/Li according to our estimation (see above). The reaction is on-going even after several days of polarization, meaning that the formed interfacial layers do not block further decomposition, at least under the given experimental conditions (ambient air, elevated temperature). Our results therefore question the stability of LLZO against high voltage cathode materials. Moreover, the current–voltage curves in Fig. 7 strongly suggest that the entire constant current regime between 1.4 V and 2.5 V is characterized by the same electrochemical process. This means that already above 1.2–1.4 V severe decomposition of LLZO takes place, which translates to a stability limit of 4.1–4.3 *vs.* Li^+^/Li at this elevated temperature.

### 4.3 Polarization at room temperature

All experiments shown so far were performed at elevated temperatures, strongly enhancing the kinetics of the occurring processes. At room temperature, at which Li-ion batteries are usually operated, the investigated LLZO decomposition might be kinetically hindered and therefore less relevant for the application of LLZO. To investigate this, a single gold electrode on a Ga:LLZO single crystal was polarized for 14 days at room temperature.

The obtained current profile shows the usual rapid decrease at the beginning of the experiment, which is followed by a current in the 0.01–0.1 nA range (approx. 0.1–1.2 μA cm^−2^ with respect to the microelectrode) for the rest of the polarization time (Fig. S4†). Analogue to the experiments above, LA-ICP-MS was used to investigate the microelectrode after polarization (Fig. S5†), revealing a significant difference to reference electrodes and thus confirming that Li-depletion occurred during the experiment. As expected, the effects induced by the polarization are significantly less pronounced at room temperature, especially considering the long polarization time. For the sample layers directly beneath the electrode (*i.e.*, the first ablation pass of the LA experiment), 82% of the initial Li-content was measured. This corresponds to a total amount of transported Li-ions of 0.130 nmol. Similar amounts were reached at 350 °C (set temperature) already after 30 min. In Table 1, the results of the analysis are compared to the amount of charge carrier transport derived from the measured current. The values are in the same order of magnitude, once more showing that the results of the LA-ICP-MS analysis are reasonable. Overall, the experiment confirms that significant LLZO decomposition occurs even at room temperature, further questioning the long-term stability of LLZO with high voltage cathode materials.

**Table 1:** Total amount of transported Li-ions during electrode polarization at room temperature (2 V voltage, 14 days polarization time, 100 μm electrode diameter), determined *via* LA-ICP-MS as well as DC measurements. The Li-ion depletion observed *via* LA agrees reasonable with the currents measured during the electrode polarization

	Total amount of transported Li^+^
LA-ICP-MS	0.130 nmol
DC	0.423 nmol
Ratio	30.1%

## 5. Conclusion

The electrochemical stability behaviour of LLZO single crystals was investigated using field stress experiments in ambient air and subsequent electrochemical, chemical, and structural analysis. Different combinations of macro- and microscopic ionically blocking Au electrodes as well as various locally resolved analysis techniques including microelectrode EIS, LIBS, LA-ICP-MS, and microfocus XRD were used for the experiments. These experiments indicate that LLZO decomposes at 4.1–4.3 *vs.* Li^+^/Li at elevated temperature (approx. 300 °C). The decomposition leads to the deposition of Li_2_CO_3_ or other Li-containing salts (LiOH, Li_2_O) at the cathodic side due to the reduction of O_2_ from air in presence of CO_2_ and H_2_O. Beneath the anode (positively polarized), La_2_Zr_2_O_7_ and La_2_O_3_ are formed due to the oxidation of O^2−^, potentially accompanied by the formation of Li-depleted LLZO (Li_7−2*x*_La_3_Zr_2_O_12−*x*_) as intermediate product. Most likely not only Li^+^ but also substantial O^2−^ conduction is involved in the decomposition process, which is on-going even several days after polarization and reaches several μm deep into the material (*i.e.*, no blocking interfacial layer is formed). Interestingly, also at room temperature significant Li-depletion can be observed, questioning the long-term compatibility of LLZO with high voltage cathode materials.

## Author contributions

Stefan Smetaczek: conceptualization, methodology, investigation (polarization experiments, ME-EIS LIBS, LA-ICP-MS, ICP-OES), formal analysis, visualization, writing – original draft. Eva Pycha: investigation (polarization experiments, LA-ICP-MS), formal analysis. Joseph Ring: investigation (polarization experiments, ME-EIS), formal analysis. Matthäus Siebenhofer: investigation (XRD). Steffen Ganschow: investigation (LLZO synthesis). Stefan Berendts: investigation (LLZO synthesis). Andreas Nenning: methodology. Markus Kubicek: methodology, funding acquisition. Daniel Rettenwander: conceptualization, resources, funding acquisition. Andreas Limbeck: resources, writing – review & editing, supervision. Jürgen Fleig: conceptualization, methodology, writing – review & editing, resources, funding acquisition, supervision.

## Conflicts of interest

There are no conflicts to declare.

## Supplementary Material

TA-009-D1TA02983E-s001
