# Supplementary material for: Investigating the electrochemical stability of Li7La3Zr2O12 solid electrolytes using field stress experiments
Source: J Mater Chem A Mater. 2021 Jun 17;9(27):15226–37. doi: 10.1039/d1ta02983e (PMC8279110; doi:10.1039/d1ta02983e)
Supplement: TA-009-D1TA02983E-s001 [file TA-009-D1TA02983E-s001.pdf]

Electronic Supplementary Material (ESI) for Journal of Materials Chemistry A.
This journal is © The Royal Society of Chemistry 2021

SUPPORTING INFORMATION

for

Investigating the Electrochemical Stability of $Li_7La_3Zr_2O_{12}$ Solid Electrolytes using Field Stress Experiments

Stefan Smetaczek[1], Eva Pycha[1], Joseph Ring[1], Matthäus Siebenhofer[1], Steffen Ganschow[2], Stefan Berendts[3], Andreas Nenning[1], Markus Kubicek[1], Daniel Rettenwander[4,5,6], Andreas Limbeck[1], Jürgen Fleig*[1]

[1]Institute of Chemical Technologies and Analytics, TU Wien, Austria

[2]Leibnitz-Institut für Kristallzüchtung, Berlin, Germany

[3]Institute of Chemistry, TU Berlin, Germany

[4]Department of Material Science and Engineering, NTNU Norwegian University of Science and Technology, Trondheim, Norway

[5]International Christian Doppler Laboratory for Solid-State Batteries, NTNU Norwegian University of Science and Technology, Trondheim, Norway

[6]Graz University of Technology, Institute for Chemistry and Technology of Materials (NAWI Graz), Graz, Austria

*Corresponding author: Juergen.Fleig@tuwien.ac.at

CONTENT:

1. Instrumental settings chemical analysis
   - ICP-OES
   - LIBS
   - LA-ICP-MS

2. Additional figures polarization of stripe electrodes
   - Current profile
   - Exemplary impedance spectra of microelectrode EIS measurements

3. Additional figures polarization of microelectrodes
   - Current profile of a constant voltage polarization experiment at elevated temperature
   - Current profile of a constant voltage polarization experiment at room temperature
   - LA-ICP-MS analysis after a constant voltage experiment performed at room temperature

1. Instrumental settings chemical analysis

Table S1: Instrumental parameters for bulk analysis via inductively coupled plasma - optical emission spectroscopy (ICP-OES)

**ICP-OES instrumentation**	**Thermo iCAP 6500 RAD**	
RF power	1200 W	
Radial observation height	12 mm	
Plasma gas flow (Ar)	12 l min^{-1}	
Nebulizer gas flow (Ar)	0.6 l min^{-1}	
Auxiliary gas flow (Ar)	0.8 l min^{-1}	
Integration time	5 s	
Replicates per sample	5	
Purge pump rate	1.6 ml min^{-1}	
Sample flow rate	0.8 ml min^{-1}	
Analytical wavelengths		
Eu (Internal standard)	281.396 nm (□)	381.967 nm (△)
Ga	417.206 nm*△	
La	333.749 nm□	412.323 nm*□
Li	610.362 nm△	670.784 nm*△
Ta	240.063 nm*□	268.517 nm*□
Zr	339.198 nm□	343.823 nm*□

* used for quantification
□/△ normalized to Eu 281.396/381.967 nm signal

Table S2: Instrumental parameters laser induced breakdown spectroscopy (LIBS) experiments

**LIBS instrumentation**	**J200**
Laser	266 nm Nd:YAG
Pulse duration	5 ns
Output energy	1.5 mJ
Beam diameter	100 µm
Scan speed	0.1 mm s^{-1}
Repetition rate	10 Hz
Beam geometry	circular
Spectrometer type	Czerny-Turner
Detection channels	6
Gate delay	0.5 µs
Gate width	1.05 ms
Atmosphere	Ar

Table S3: Instrumental parameters laser ablation - inductively coupled plasma - mass spectrometry (LA-ICP-MS) experiments

**Laser ablation system**	**New Wave 213**
Average fluence	3.0 J cm^{-2}
Laser diameter	60 µm
Scan speed	15 µm s^{-1}
Repetition rate	20 Hz
Carrier gas flow (He)	0.6 L min^{-1}
Make-up gas flow (Ar)	0.8 L min^{-1}
**ICP-MS instrumentation**	**Thermo iCAP Q**
Auxiliary gas flow (Ar)	0.8 L min^{-1}
Cool gas flow (Ar)	14 L min^{-1}
Dwell time per isotope	10 ms
RF power	1550 W
Cones	Ni
Mass resolution	$m/\Delta m = 300$
Measured isotope	^{7}Li, ^{90}Zr, ^{138}La, ^{181}Ta

2. Additional figures polarization of stripe electrodes

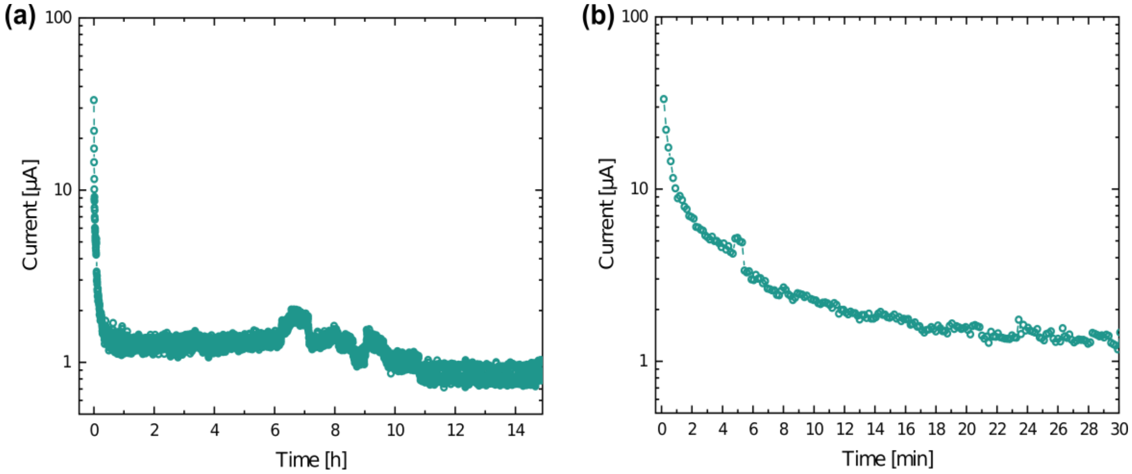

Figure S1: Current measured during polarization (3 V) via stripe electrodes at 400 °C (set temperature). While (a) shows the entire experiment, (b) shows a magnified view of the first 30 min. A rapid current drop in the first few minutes of the experiment is observable, which is followed by a stabilization of the current in the 1 µA range. Minor current fluctuations are visible in the time interval between 6 and 11 h, most likely caused by Li_2CO_3 and/or O_2 formation impacting the electrode/electrolyte interface.

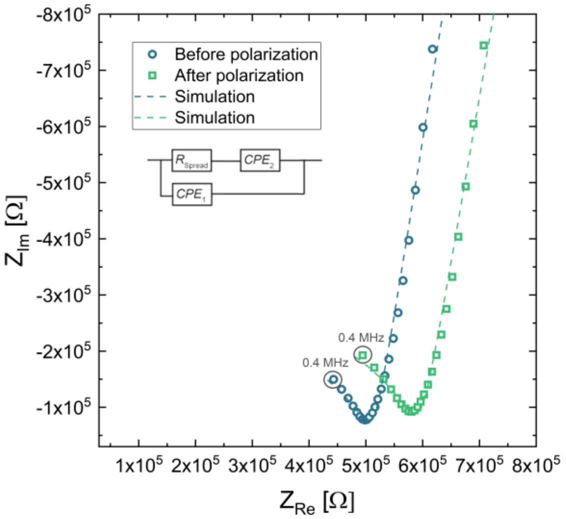

Figure S2: Exemplary impedance spectra of microelectrode EIS measurements performed at room temperature on the same electrode before and after a polarization experiment. The corresponding fits (dashed lines) are based on the shown equivalent circuit. The high frequency arc is strongly affected by the applied field stress, which corresponds to changes in the charge transport properties of the sample.

3. Additional figures polarization of microelectrodes

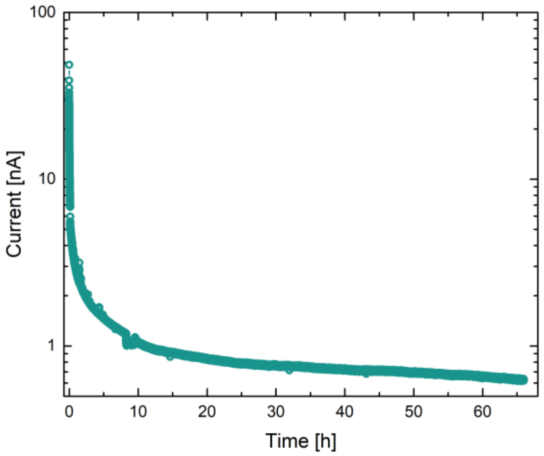

Figure S3: Typical current profile for a microelectrode constant voltage polarization experiment (Ta:LLZO single crystal, 2 V voltage, 66 h polarization time, 350 °C set temperature, 100 µm electrode diameter). The measured current decreases over time but stays above 0.5 nA even after several days of polarization, indication an on-going decomposition reaction.

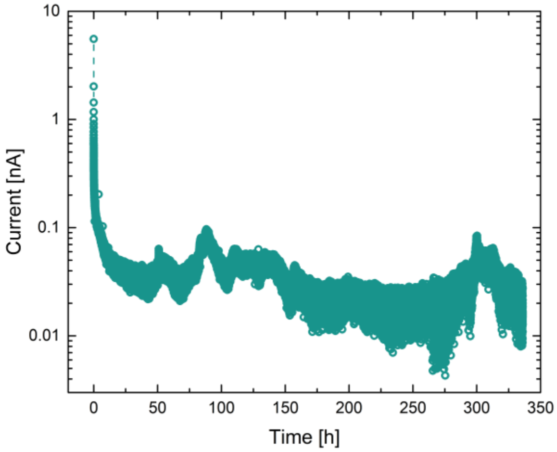

Figure S4: Current profile for a microelectrode constant voltage polarization experiment performed at room temperature (Ga:LLZO single crystal, 2 V voltage, 14 day polarization time, 100 µm electrode diameter). After a rapid decrease at the binning of the polarization, the current stays in the 0.01 – 0.1 nA range for most of the experiment. Significant current fluctuations are visible, indicating changes of the electrode/electrolyte interface caused by the polarization (e.g., O_2 formation leading to gas bubbles beneath the electrode).

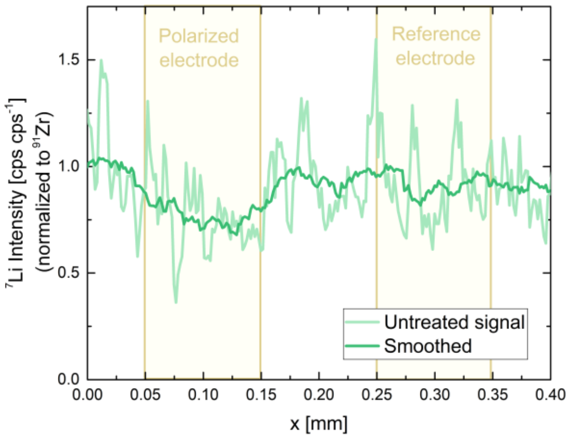

Figure S5: Normalized Li signal of a LA-ICP-MS analysis conducted after a constant voltage experiment performed at room temperature (Ga:LLZO, 2 V voltage, 14 days polarization time, 100 µm electrode diameter). In addition to the untreated signal, smoothed data (obtained by moving averaging) is shown for better visualization of the results. A significant difference between polarized and reference electrode is visible, which is confirmed by comparison with further untreated electrodes (not shown).
